# Tissue clearing of human iPSC-derived organ-chips enables high resolution imaging and analysis†

**DOI:** 10.1039/d2lc00116k

**Published:** 2022-10-07

**Authors:** Briana N. Ondatje, Samuel Sances, Michael J. Workman, Clive N. Svendsen

**Affiliations:** Cedars-Sinai Board of Governors Regenerative Medicine Institute Los Angeles CA USA clive.svendsen@cshs.org

## Abstract

Engineered microfluidic organ-chips enable increased cellular diversity and function of human stem cell-derived tissues grown *in vitro*. These three dimensional (3D) cultures, however, are met with unique challenges in visualization and quantification of cellular proteins. Due to the dense 3D nature of cultured nervous tissue, classical methods of immunocytochemistry are complicated by sub-optimal light and antibody penetrance as well as image acquisition parameters. In addition, complex polydimethylsiloxane scaffolding surrounding the tissue of interest can prohibit high resolution microscopy and spatial analysis. Hyperhydration tissue clearing methods have been developed to mitigate similar challenges of *in vivo* tissue imaging. Here, we describe an adaptation of this approach to efficiently clear human pluripotent stem cell-derived neural tissues grown on organ-chips. We also describe critical imaging considerations when designing signal intensity-based approaches to complex 3D architectures inherent in organ-chips. To determine morphological and anatomical features of cells grown in organ-chips, we have developed a reliable protocol for chip sectioning and high-resolution microscopic acquisition and analysis.

## Abstract 2

Optimization of SCALE protocol for organ-chips and organ-chip sections for improved visualization.

## Graphical abstract

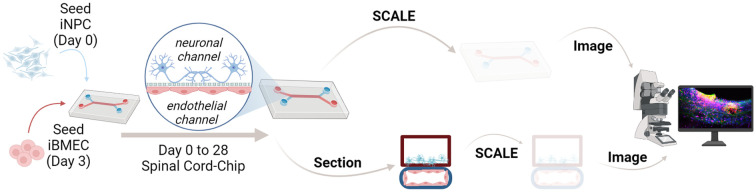

## Introduction

High-throughput screening approaches that utilize sparsely plated homogenous monolayers of cells in multi-well culture plates are a proven tool in image-based biomedical science; however, the fidelity of such cultures is limited. The combination of induced pluripotent stem cell (iPSC) technology with microengineered culture platforms known as “organ-chips” enable highly functional multi-cellular mimics of organ function.^1,2^ Organ-chips representing gut,^3^ heart,^2^ and brain^4–6^ exhibit increased tissue growth and development within this 3D structure, which can be attributed to the increased organ-specific fidelity and functions reported from these systems. However, the 3D nature of advanced organ-chip architecture presents imaging challenges that are similar to staining and imaging of tissue *in vivo*, namely, sample focal distance, variance in refraction within the tissue, inefficient antibody penetrance, and decreased light penetrance due to tissue opacity.

To circumvent these challenges, multiple tissue clearing methods have been developed, for instance CLARITY (Clear Lipid-exchanged Acrylamide-hybridized Rigid Imaging/Immunostaining/*In situ* hybridization-compatible Tissue-hYdrogel), an electrophoretic tissue clearing technique, that is fast acting and can clear tissue in a matter of days.^7–10^ These methods increase optical access for microscopy to enable spatial protein analysis of intact tissue and even whole organisms.^11^ However, they are not applicable to organ-chips as they employ organic solvents that are destructive to siloxane-based polymers such as polydimethylsiloxane (PDMS), a common material for the construction of organ-chip systems. To overcome these limitations, alternative aqueous-based solvent techniques can be utilized.^8^

Here, we adapted a hyperhydration clearing technique, ScaleS (SCALE with sorbitol)^12^ to visualize cell-specific marker production within the neuronal compartment of our human Spinal Cord-Chip system (SC-Chip),^5^ which is established using induced pluripotent stem cells (iPSCs) differentiated into spinal neural progenitor cells (spNPCs) seeded into the top channel and brain microvascular endothelial cells (BMECs) seeded into the bottom channel. The resulting organ-chip contains a thick neural channel, necessitating tissue clearing to aptly visualize the cells with antibody staining. This tissue clearing protocol is highly applicable to organ-chips for three reasons: the urea-based clearing solutions were compatible with PDMS, the final clearing solution closely matches the refractive index of 1.44 of PDMS *vs.* 1.33 of phosphate-buffered saline (PBS), and due to low viscosity, the series of clearing steps could be perfused with minimal disruption to the tissue of interest. By condensing the 1–2 week process,^12^ perfusion of clearing and staining solutions enabled rapid processing of intact chips in 3 days. To visualize protein production within cultured cells, we also devised methods to section, clear, and stain 200 μm cross-sections of the organ-chips, enabling population analysis of intact cells *in vitro*. Three dimensional multicellular cultures in organ-chips enhance faithful modeling of the *in vivo* milieu, and this modified protocol to improve cell and protein visualization provides the scientific community with new avenues to address complex questions.

## Results

### Spinal cord-chips generate thick neural layer

**Fig. 1:**
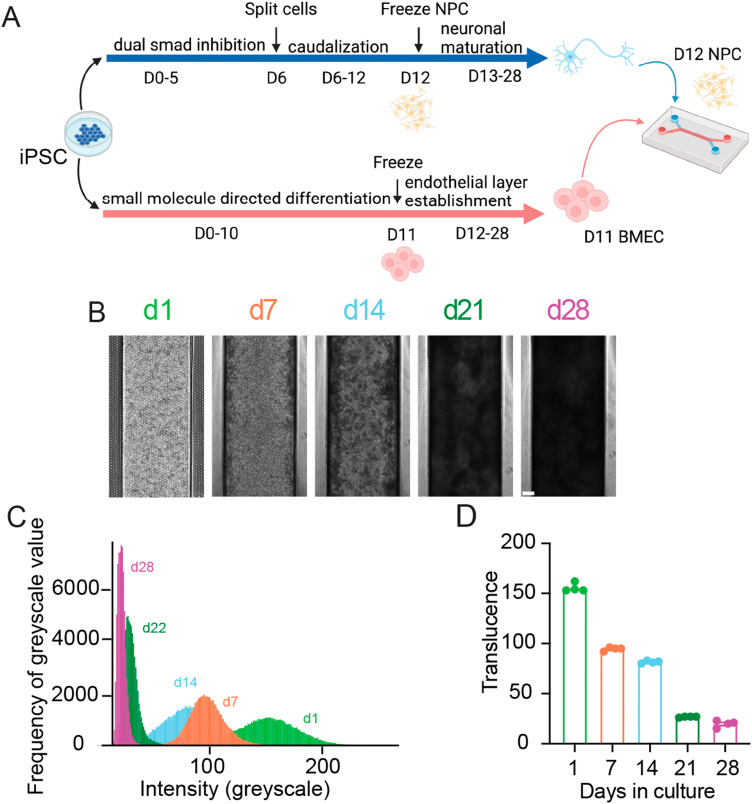
SC-Chips neural tissue increases in opacity overtime. (A) Schematic of SC-Chip culture. Human spNPCs and BMECs are cultured from iPSCs, with the spNPCs seeded on the top channel, and the BMECs seeded on the bottom channel, cultured under flow. (B) Tissue growth and opacity increase over time (scale bar = 200 μm). (C) Histogram of greyscale values from a single chip over time. (D) Average translucence of multiple chips over days in culture.

To generate organ-chips to test tissue clearing and imaging, we developed SC-Chips based on established protocols,^5^ but with some optimizations to reduce neural detachment that included gravity fed flow through the channels and an increased seeding density of neural cells. iPSC lines from control individuals (EDi028-A and 02iCTR) were previously generated^13^ and used to derive both spNPCs and BMECs. A 12 day directed differentiation protocol was used to generate spinal neural progenitors, which were then cryopreserved for a spNPC bank. The iPSCs were differentiated into BMECs using an 11 day directed differentiation protocol, with subsequent cryopreservation for a BMEC bank. The spNPCs were thawed and seeded into the top channel at day 0, and BMECs were thawed and seeded in the bottom channel on day 3 of the experiment (Fig. 1).

Gravity flow was introduced on day 4. Cells were cultured for 28 days, under a continuous gravity flow at a rate of 6 μl per hour. This flow is in contrast to the established protocol that used a static manual feed of 50 μL every other day, and we found that continuous gravity flow led to reduced neural cell detachment and hence a thicker neural layer. At regular one-week intervals, SC-Chips were imaged live under phase contrast (Fig. 1B). Over time, the contrast of the neuronal channel increased, indicating increased opacity and neural tissue thickness (Fig. 1B). To quantify the changes in opacity overtime, histograms were generated to show the shift in opacity from a single chip (Fig. 1C). Measuring the average translucence across four chips showed a significant change over days in culture (Fig. 1D).

### Organ-chip clarification provides cell and protein visualization

**Fig. 2:**
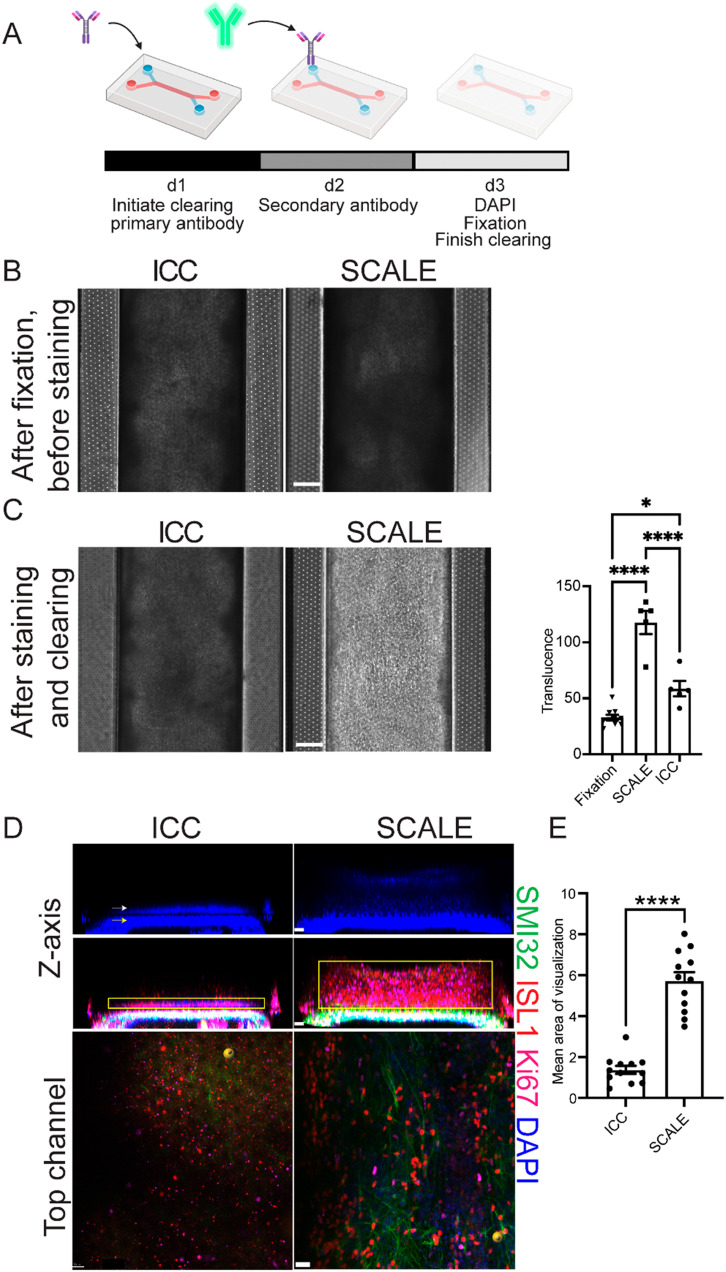
Tissue opacity is effectively cleared using the SCALE method on chips. (A) Schematic of the SCALE process modified for organ-chip use. (B) Comparison of ICC *vs.* SCALE phase imaging d28 (scale bar = 200 μm). (C) Phase imaging of ICC *vs.* SCALE after undergoing their respective staining protocols, and quantification of opacity changes from phase images of chips processed using either ICC or SCALE (scale bar = 200 μm) (*n* = 10 chips for fixation, *n* = 5 chips for SCALE and ICC, one-way ANOVA adjusted *P* values are *p* < 0.0001 fixation *vs.* SCALE, *p* = 0.0131 fixation *vs.* ICC, and *p* < 0.0001 SCALE *vs.* PBS, error bars = SEM). (D) Confocal images of ICC and SCALE chips from Fig 2B, top panels indicate DAPI. White and yellow arrows highlight cells in the top channel and bottom channel, respectively. The middle panels show non-phosphorylated neurofilament heavy chain (SMI32) and islet1 (ISL1)-positive spinal motor neurons, as well as Ki67-postive proliferating cells in the top neuronal channel. Yellow box indicates area quantified for E. Bottom panel is a top-down view of the neuronal channel. All images shown in B–D are taken from the same chips. (E) Quantification of area of visible stain in the top channel (*n* = 12 sites per condition, 4 individual chips per condition, scale bar *z*-axis = 50 μm, scale bar top channel = 30 μm, *p* < 0.0001, unpaired *t*-test, error bars = SEM).

In order to reduce opacity after fixation, the SCALE method traditionally used to clear brain tissue^12^ was optimized for organ-chips. The protocol was shortened for this new application, with organ-chips incubated in each solution for approximately a tenth of the originally published duration for mouse brain (Fig. 2A and S1†). Solutions were introduced by slowly adding them through both channels using a pipette, followed by incubation. To determine the extent to which SCALE tissue clearing improved sample resolution over standard methods, the SCALE method was compared to a step-matched standard immunocytochemistry (ICC) protocol (Fig. 2A and S1,† and Materials and methods). At culture endpoint (d28), both chips post-fixation were similarly opaque, indicating comparable tissue thickness (Fig. 2B). The endpoint of the neuronal channel results in a pigmented tissue layer, whereas, the BMEC channel has layers of translucent flat cells (Fig. S2†). Upon completion of the SCALE protocol, translucence by phase imaging was significantly increased compared to fixation before SCALE and to the ICC condition (Fig. 2C). To identify different cell populations in the intact SC-Chips, florescent confocal imaging was conducted spanning both top and bottom channels using neuronal and BMEC-specific antibodies. Reconstructed 3D images representing an optical cross-section of the two channels revealed thick neural tissue along the top channel (Fig. 2D). Non-phosphorylated neurofilament heavy chain (SMI32) and Islet1 (ISL1)-positive staining showed the presence of spinal motor neurons throughout the tissue thickness (Fig. 2D). The signals in the ICC-perfused chip were very weak, likely due to inefficient penetration of the staining solutions in the top channel, thereby rendering it difficult to effectively acquire data (Fig. 2D top). Top-down view of the SCALE condition resolved neurite morphology that was completely occluded in the ICC condition (Fig. 2D bottom). The mean area for visualized antibody signal was significantly increased in the SCALE-treated chips when compared to the standard ICC-treated chips (Fig. 2E). These experiments show that SCALE greatly improves solution penetration and can be used effectively to stain and image thick chip tissues (Fig. S3† videos).

### Organ-chip sectioning further enhances cell and protein visualization

**Fig. 3:**
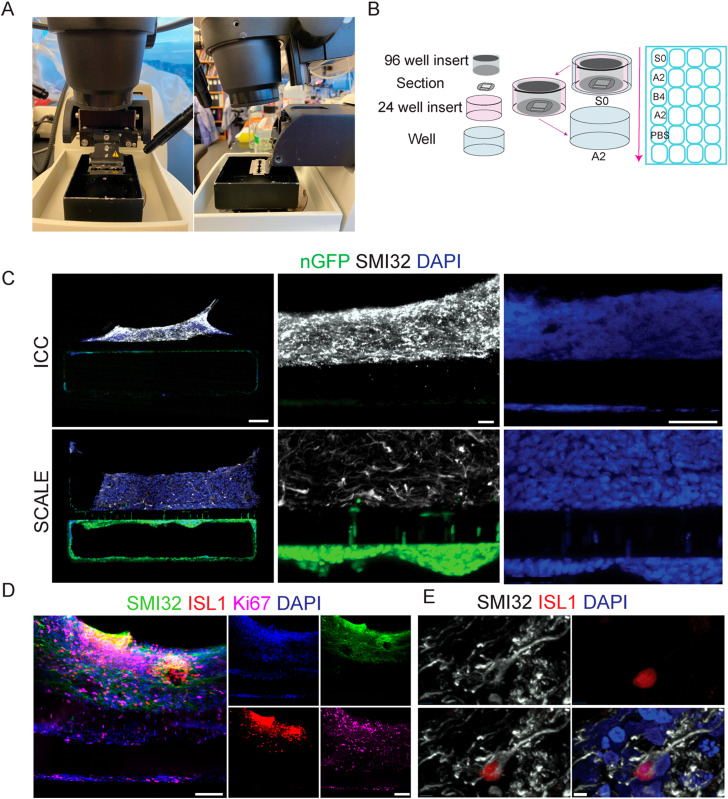
SCALE of chip sections enables high resolution microscopy. (A) Representative image of the blade setup of the vibratome for PDMS chip sectioning. (B) Schematic of staining process for sections in 24 well plate. (C) ICC *vs.* SCALE sections showing SMI32 (white) for spinal motor neurons and nGFP (green) for BMECs, with zoomed in images of SMI32, GFP, and DAPI (scale bar = 100 μm left, 20 μm center, scale bar = 50 μm right). (D) Image at 20× shows imaging capacity for multiple cell markers in 4 wavelengths: 488, 594, 647, 405 (scale bar = 100 μm). (E) Image at 60× oil objective from section using tissue clearing protocol shows ability to decipher individual cell morphology with SMI32 (white), ISL1 (red), and DAPI (blue) (scale bar = 5 μm).

While clearing was effective, increased distance to the intact sample due to PDMS limited imaging to only 10×. Additionally, the chips deteriorated after only two weeks while stored in PBS at 4 °C. To visualize chip data at higher magnification, classical histology tissue sectioning methods were next tested on the PDMS-encased tissues. Vibratome sectioning of fixed organ-chips at 200 μm thickness was found to cause minimal sample disruption. Blade placement on the vibratome is critical for successful sectioning of the PDMS chip. During setup, a full blade must be placed in the blade holder, with the blade protruding slightly out from the arm (Fig. 3A). Multiple protocols addressing technical movement and physical placement of the sections once under the ICC protocol were tested. Handling the chip sections proved difficult for solution changes as well as tissue stability; consequently, a new method was developed to move sections during processing, in which the section is contained within a 24 well insert thereby minimizing tissue disruption during the staining protocol (Fig. 3B). The initial sections stained using standard ICC techniques showed an inconsistent degree of antibody penetrance, possibly due to hinderance from the PDMS causing the sections to float on the surface (data not shown). To alleviate this, the staining method was further modified to secure sections with a 96 well insert on top of the section.

We next tested sections of SC-Chips using both standard ICC solutions as well as SCALE solutions (Materials and methods). For this experiment, BMECs stably expressing nuclear green fluorescent protein (nGFP) were seeded in the bottom channel. Signal was visible for both endogenous GFP fluorescence and immunocytochemistry for SMI32 (Fig. 3C). While both protocols show specific SMI32 staining, there appeared to be more non-specific background with ICC compared to the SCALE treatment (Fig. 3C). Tissue expansion was observed (low magnification) under the SCALE process, which may contribute to a reduced background by permitting better rinses. There is clear endogenous fluorescent signal from the nGFP-BMECs, which was stronger in the SCALE-treated sections compared to ICC (Fig. 2C). Of note, sections shown were taken from consecutive sections 200 μm apart in which cells are expected to be the most similar to one another in terms of composition as well as density. Comparing only DAPI, a more defined appearance of the nuclei is possible with the SCALE staining protocol as opposed to standard ICC (Fig. 3C). Additionally, imaging with a 20× objective was possible with sectioned chips, and co-labelling with multiple antibodies demonstrated motor neuron populations, neurofilament extensions, and dividing cells (Fig. 3D). Critically, 60× objective visualization of individual cell morphology was also accomplished, which was previously not possible under whole intact chip imaging conditions (Fig. 3E). This new method permits clear discrimination of individual DAPI-positive cells and interrogation of the morphology of individual ISL1-positive neuronal cells.

## Discussion

### Application of clarification of organ-chips

Organ-chips are increasingly being used as a tool to improve disease modeling and translational medicine, providing a platform to study diseases and assess their potential drug candidates.^14–17^ Combining microphysiological systems and human iPSCs avoids limitations of animal-based studies and can permit more faithful modeling of human disease.^2–5,15^ The co-culture of multiple cell types within the organ-chip and the introduction of controlled laminar flow are predicted to induce cell maturity of iPSC-derived cells by introducing them to a more *in vivo*-like environment.^4–6,18^ While developing a blood brain barrier-chip model, it was discovered that introducing continuous perfusion of media led to greater maturity of BMECs compared to a static culture.^4^ Furthermore, development of the SC-Chip model showed that co-culture with iPSC-derived BMECs led to enhanced motor neuron maturity and survival.^5^ However, these organ-chips created a challenge when attempting to visualize the cellular contents of the chip through ICC due to the increased tissue density and opacity. The conventional staining techniques utilizing standard ICC solutions do not provide the resolution needed for these larger tissues due to issues with antibody penetrance and light penetrance into the deeper parts of the chip. Antibody penetrance issues can be solved by weeks of incubation; however, this is not ideal for time-sensitive experiments and results. The light penetrance issue cannot be resolved with standard immunocytochemistry practices due to inherent pigments and other molecules that scatter light.^12^ A solution to curtail these problems was to apply tissue clearing and sectioning to organ-chips.

Organic solvents, used in traditional tissue clearing, present a challenge to organ-chip users in that the majority of organ-chips are based on PDMS structures that should deteriorate in the presence of these organic solvents. Alternatively, hyperhydration techniques, such as ScaleS,^12^ have been developed that utilize urea-based and other chemical agent solvents to optically clear tissues.^8,19^ Here, the timing of the established ScaleS protocol was reduced and proportioned to a micro scale. It was found that organ-chips can effectively be cleared and visualized for cellular characterization in just three days.

The SC-Chip showed increased tissue density over the course of a 28 day culture, which resulted in decreased light penetrance and translucence over time (Fig. 1). To circumvent this, tissue clearing was tested and led to a dramatic increase in the translucence of the tissue (Fig. 2). This reduction is caused by the urea-based solutions of the SCALE protocol,^8^ whose mechanism is not fully understood. In choosing and optimizing the SCALE protocol, a few important considerations were taken into account: the size of the tissue to be explored and how much disruption of the tissue can be expected from the clearing protocol. SC-Chips contain thick yet delicate tissue that require as little disruption as possible since the tissue develops upon itself without any added scaffold.^5^ Therefore, it is imperative to avoid as much dehydration of the tissue as possible; this is one of the reasons the SCALE protocol was determined to be an optimal clearing method to test and implement on the organ-chips. Another consideration is the refractive index of the organ-chip's material, in order to choose the best matching solution, particularly when imaging the whole intact chip is the ultimate goal. For this reason, the SCALE S4 solution was chosen as it matches the refractive index of PDMS.^12^ The SCALE protocol takes ten days for a tissue that is 1–2 mm thick, yet SC-Chips have a tissue thickness of only about 400 μm after tissue expansion post-stain. Consequently, a protocol was designed to reach translucence and subsequently acquire fluorescent imaging after only 3 days (Fig. 2A and D). Using this modified SCALE protocol, images were successfully acquired from both the top and bottom channels of the chip. Imaging does indicate substantial expansion of the tissue in the SC-Chip having undergone the SCALE process (Fig. 3C). This improves the ability to resolve tissues as well as improves penetration of antibody, which is the basis of expansion microscopy.^20,21^ Additional measures currently being tested to further improve the quality of the stain are to increase the concentration of Triton-X in the SCALE A2 solution to 1%, which is a more aggressive disruption of the membrane to allow increased antibody penetrance, as well as incubation at 37 °C, as described in the original SCALE protocol, which may improve diffusion and penetration of the clearing solutions and antibodies.^12^

### Advantages of organ-chip sectioning

While tissue clearing was effective at reducing the opacity on whole intact organ-chips, the protocol remained limited to a 10× objective and the antibody signal continued to not penetrate the inner most depths of the neuronal tissue channel. Utilizing a vibratome and conventional histology tools, the PDMS organ-chip sections can be obtained and undergo the modified organ-chip SCALE or standard ICC protocol. When the blade was setup in the manner described in the manual, the tissue detached from the PDMS upon sectioning. Acquiring sections from the PDMS chip requires a specific blade setup that allows the tissue inside the chip to be distanced enough from the motor which in turn keeps the tissue intact during the mechanical process of sectioning. The developed method for organ-chip sectioning addresses penetrance difficulties by increasing the mass to volume ratio and allowing the solutions to interact with the tissue from the top and bottom. This now permits the visualization of the cellular morphology and phenotype within the tissues. Of note, staining chip sections using a standard ICC protocol was complicated by increased tissue detachment from the PDMS, compared to when using the SCALE protocol. It is unclear what led to this difference, but it may be related to different viscosities of the staining solutions in the two protocols. While less sections were available to process by ICC, we were able to show that cells could be visualized by immunocytochemistry and for endogenous fluorescence from the nGFP BMECs. However, in comparison to SCALE-processed sections, ICC-processed sections showed more non-specific antibody staining and greatly reduced visualization of GFP. A clear limitation of the intact chip is that each chip can only provide a sample for one antibody run. Sectioning one organ-chip yields approximately 48 sections, which provides the opportunity for multiple rounds of antibody analyses of cell populations and protein production. A further limitation is that the intact SC-Chip only retained decent tissue quality for imaging for about two weeks before the tissue began to deteriorate, whereas organ-chip sections retained a good quality for staining for up to a year post-fixation when held in PBS at 4 °C. Critically, higher magnification imaging is obtainable following organ-chip sectioning, permitting clear visualization of individual cells as well as their neural extensions and intracellular protein localization. A caveat to 60× imaging in this protocol was that an oil immersion lens was used. Using a glycerol objective would provide a more compatible refractive index to further improve resolution. Finally, as sections remain mounted in a wet solution throughout the imaging process, the oil immersion lens can easily displace the coverslip off the section; hence optimization for mounting media should be considered.

## Conclusions

This organ-chip clearing, staining, and sectioning method is advantageous to image thicker tissue and could prove beneficial for systems such as the gut where substantial growth occurs within a couple of weeks. Additionally, the higher resolution will improve capabilities of deciphering separate populations of cells within intact cultures, such as full neural vascular units containing upwards of five cell types. Finally, these techniques now provide the groundwork for the ability to scrutinize synaptic formation or neuromuscular junctions within the organ-chip platform. Organ-chips are an ideal *in vitro* model to recapitulate the *in vivo* multicellular environment. These developed methods now provide the ability to deeply analyze multicellular interactions, which can enhance disease modeling and drug screening by the scientific community.

## Materials and methods

### Culturing SC-Chips

Microfluidic chips (Emulate Inc.) were used to co-culture iPSC-derived spinal motor neurons and BMECs for 28 days. Briefly, two donor control iPSC lines (EDi028-A or 02iCTR) were generated using non-integrating virus by the Cedars-Sinai iPSC Core, which have been previously characterized.^13^ A 12 day directed differentiation protocol utilizing dual smad inhibition, CHIR, retinoic acid, and smoothened agonist in which cells are expanded from 1 well into 6 wells on day 6 was used to generate (under IRB # for iPSC maintenance and differentiation 21505, IRB # for chip work 49203) spinal motor neuron cultures, which were then cryopreserved for a spNPC bank on day 12. The 02iCTR line was differentiated into BMECs using an 11 day directed differentiation utilizing beta-mercaptoethanol, CHIR, retinoic acid, and FGF, with subsequent cryopreservation for a BMEC bank. The iPSC-derived spNPCs were thawed and seeded into the top channel at day 0 of the organ-chip experiment, with BMECs thawed and seeded on day 3 of the experiment. Gravity flow was introduced on day 4. Some organ-chip sectioning experiments used a 02iCTR line transfected to stably produce nGFP. Imaging was performed once weekly for the duration of the culture on a Nikon Biostudio-T using a 1.6× objective. On d28 organ-chips were fixed and stored until ready for staining. Illustrations created with https://www.BioRender.com.

### Standard immunostaining of whole organ-chips

Organ-chips were fixed with 4% paraformaldehyde (PFA) in PBS. Using a P200 pipette, 100 μL of the staining solutions were taken up in which 50 μL of the solution was flowed through each channel to flush out prior solution, leaving 50 μL to incubate within the chip. Chips were washed with PBS 3× 5 min and stored at 4 °C until ready to stain. For staining, chips were washed in PBS for 4.5 h to match incubations with the SCALE protocol. Chips were blocked in a solution of 5% normal donkey serum (NDS), 1% bovine serum albumin (BSA), and 0.5% Triton-X for 1 h. Then they were washed in 0.5% Triton-X in PBS for 30 min. Primary antibodies in blocking solution were added to the chips overnight at 4 °C. The following primary antibodies were used for immunocytochemistry: mouse mAb to SMI32 (Biolegend, 801701, 1 : 1000), goat polyAb to Islet-1 (R&D Systems, AF1837, 1 : 500), rabbit Ki67 (abcam, ab27619, 1 : 200). Samples were then washed in 0.5% Triton-X solution in PBS 2× 10 min. Secondary antibodies were mouse IgG conjugated to Alexa Fluor 488, and goat IgG conjugated to Alexa Fluor 594 (Invitrogen; A-21202, A-21207, respectively), used at 1 : 1000 in a 1% BSA and 0.5% Triton-X solution for 24 h at room temperature. Following the secondary antibodies, DAPI was added 1 : 1000 for 5 min then subsequently washed with 0.5% Triton-X in PBS for 1 h 20 min and then left in PBS until ready to image.

### SCALE solutions

Fresh solutions were made in PBS with their respective concentrations of urea, Triton-X, and/or glycerol and the other components included.^12^ S0: d-sorbitol 20% w/v, 5% w/v glycerol, 1 mM methyl-beta-cyclodextrin, 1 mM gamma-cyclodextrin, 1% w/V *N*-acetyl-l-hydroxyproline, 3% v/v dimethylsulfoxide, 1× PBS, ScaleA2: 10% w/v glycerol, 4 M urea, 0.1% w/v triton-X-100, 1× PBS, ScaleB4(0): 8 M urea, 1× PBS, AbScale: 0.33 M urea, 0.1% w/v Triton-X-100, 1× PBS, AbScale Rinse: 2.5% w/v Tween-20, 0.05% w/v BSA, 0.1× PBS, S4: 40% w/v d-sorbitol, 10% w/v glycerol, 4 M urea, 0.2% Triton-X-100, 15% v/v dimethylsulfoxide, 1× PBS.

### SCALE staining of whole organ-chips

Endpoint organ-chips were fixed in a 4% PFA in PBS at room temperature for 1 h. Samples were then stored in PBS at 4 °C until ready to stain. For the SCALE and staining process, SCALE S0 was incubated for 20 min ×3. Samples were then cleared in SCALE A2 for 40 min ×2, then SCALE B4 for 30 min ×3, and then again in SCALE A2 for 20 min ×3. After, samples were descaled in PBS for 10 min ×2 followed by immunofluorescent staining. The samples were incubated in AbSCALE solution containing the primary antibodies (listed above in immunostaining section) for 24 h at 4 °C, then were washed ×2 for 10 min with AbSCALE solution and introduced to the secondary antibodies (listed above in immunostaining section) in AbSCALE solution for 24 h. Following secondary incubation, DAPI at 1 : 1000 in AbSCALE solution was added for 5 min and subsequently washed for 10 min ×3 with AbSCALE solution followed by AbSCALE rinse ×2 for 10 min. A second fixation was then done for 20 min with 4% PFA, then washed with PBS for 10 min. Final clearing was then performed using S4 for 20 min ×3, and then samples were stored in SCALE S4 at 4 °C until ready for imaging. All solutions were incubated at room temperature unless otherwise specified. Illustrations created with https://www.BioRender.com.

### Organ-chip sectioning

Whole organ-chips were fixed with 4% PFA in PBS for 1 h. Samples were then stored in PBS at 4 °C until ready to section. The chips were cut in half at the vacuum ports and sectioned one half at a time. The excess PDMS was trimmed at the intersection of the channels, then the sides were trimmed along the side of main channel and off the top of the channels creating a long thin rectangle. Taking one of the trimmed off pieces of PDMS, ensuring it was level, the piece was mounted onto the vibratome chuck with superglue, and the chip to be sectioned was mounted onto the piece of PDMS. This method was used to raise the chip and obtain more sections. They were sectioned on a Leica VT 1200 vibrating blade microtome at 200 μm thick at an automated set rate of 0.30 mm s^−1^. Samples were then stored in PBS at 4 °C until ready to stain.

### Standard immunostaining of organ-chip sections

For staining, chips were washed in PBS for 20 min ×3, 40 min ×3, 30 min ×3, to match incubations with the SCALE protocol. Sections were blocked in a solution of 5% normal donkey serum (NDS), 1% bovine serum albumin (BSA), and 0.5% Triton-X for 20 min ×3. Then they were washed in 0.5% Triton-X in PBS for 10 min ×3. Primary antibodies in blocking solution were added to the sections overnight at 4 °C. The following primary antibodies were used for immunocytochemistry: mouse mAb to SMI32 (Biolegend, 801701, 1 : 1000), goat polyAb to Islet-1 (R&D Systems, AF1837, 1 : 500), rabbit Ki67 (abcam, ab27619, 1 : 200). Samples were then washed in 0.5% Triton-X solution in PBS 2× 10 min. Secondary antibodies were mouse IgG conjugated to Alexa Fluor 488, and goat IgG conjugated to Alexa Fluor 594 (Invitrogen; A-21202, A-21207, respectively), used at 1 : 1000 in a 1% BSA and 0.5% Triton-X solution for 24 h at room temperature. Following the secondary antibodies, DAPI was added 1 : 1000 for 5 min then subsequently washed with 0.5% Triton-X in PBS for 10 min ×5, and then again for 20 min, following these washes the sections were then washed in PBS for 10 min, and then left in PBS until ready to image.

### SCALE staining of organ-chip sections

The section was placed in one well of a 24 well plate with 500 μL of the starting solution containing a 24 well staining mesh insert. Ensuring that the section was centralized in the well, a 96 well insert cut from a Nunc 96 well mesh plate insert was placed on top of the section to assure submersion of the section in the solution. For gentle transition, the section was then transferred within the 24 well mesh while also maintaining the 96 well insert on top to the next solution in a neighbouring 24 well after incubation. For the SCALE and staining process, SCALE S0 was incubated for 1 h. Samples were then cleared in SCALE A2 for 2 h, then SCALE B4 for 1.5 h, and then again in SCALE A2 for 1 h. After, samples were descaled in PBS for 30 min followed by immunofluorescent staining. The samples were incubated in AbSCALE solution containing the primary antibodies (listed above in immunostaining section) for 24 h at 4 °C, then were washed for 10 min, twice with AbSCALE solution and introduced to the secondary antibodies (listed above in immunostaining section) in AbSCALE solution for 24 h. Following secondary incubation, DAPI at 1 : 1000 in AbSCALE solution was added for 5 min and subsequently washed for 30 min with AbSCALE solution followed by AbSCALE rinse for 10 min, twice. A second fixation was then done for 20 min with 4% PFA, then washed with PBS for 10 min. Final clearing was then performed using ScaleS4 for at least 1–2 h, and then stored in SCALE S4 at 4 °C until ready for imaging. All solutions were incubated at room temperature unless otherwise specified. Mounting was achieved by placing the section directly onto slides and then applying a drop of mounting media (SCALE S4) on top of the section, ensuring it was in the channels to avoid bubbles. A circular coverslip was then placed on top and filled in by placing a p200 pipette with mounting media near the edge of the slip and expelling until coverslip was filled in completely.

### Light penetrance measurements

Light penetrance histograms were generated from Nikon Biostudio-T images taken with a 1.6× objective and analyzed in ImageJ Software. The main channel was selected and analyzed using the histogram function taking the greyscale value of each pixel and generating frequency table of each greyscale value 0 to 255 for the selection. Histograms were overlaid and peaks were extracted for translucence measurements using RStudio. Statistics performed on Graphpad Prism 8. Time course over live culture was analyzed using an RM one-way ANOVA with Tukey's multiple comparison with an overall significance of *p* < 0.0001, d1 *vs.* d7 *p* < 0.0012, d1 *vs.* d14 *p* < 0.0005, d1 *vs.* d21 *p* < 0.0001, d1 *vs.* d28 *p* < 0.0001, d7 *vs.* d14 *p* < 0.0015, d7 *vs.* d21 *p* < 0.0001, d7 *vs.* d28 *p* < 0.0001, d14 *vs.* d21 *p* < 0.0001, d14 *vs.* d28 *p* < 0.0004, d21 *vs.* d28 *p* < 0.1219, error bars indicate SEM. Fixation *vs.* SCALE *vs.* PBS was compared using an ordinary one-way ANOVA, fixation *vs.* SCALE *p* < 0.0166, fixation *vs.* PBS *p* < 0.1461, SCALE *vs.* PBS *p* < 0.2654, error bars indicate standard error mean (SEM).

### Area of visualization measurement

Images were acquired on a Nikon A1R using the 10× objective. The area was calculated using ImageJ by specifying a box around the visible signal and taking the area measurement of that box. Statistics performed on Graphpad Prism 8 using an unpaired *t*-test, *p* < 0.0009, error bars indicate SEM.

## Author contributions

Briana Ondatje contributed to the conceptualization, methodology, investigation, formal analysis, validation, visualization, and writing of this article. Samuel Sances contributed to the conceptualization and methodology, and writing of this work. Michael Workman helped develop the methodology of the sectioning protocol. Clive N. Svendsen contributed to the conceptualization, methodology, investigation, formal analysis, and writing of this article.

## Conflicts of interest

Cedars-Sinai owns a minority stock interest in Emulate, the company that produces the study's microfluidic organ-chips. An officer of Cedars-Sinai also serves on Emulate's Board of Directors. Emulate provided no financial support for this research. Cedars-Sinai and Emulate have patents filed related to this work.

## Supplementary Material

LC-022-D2LC00116K-s001

LC-022-D2LC00116K-s002
