# Supplementary material for: Tissue clearing of human iPSC-derived organ-chips enables high resolution imaging and analysis
Source: Lab Chip. 2022 Oct 7;22(21):4246–55. doi: 10.1039/d2lc00116k (PMC9595176; doi:10.1039/d2lc00116k)
Supplement: LC-022-D2LC00116K-s001 [file LC-022-D2LC00116K-s001.pdf]

Electronic Supplementary Material (ESI) for Lab on a Chip.
This journal is © The Royal Society of Chemistry 2022

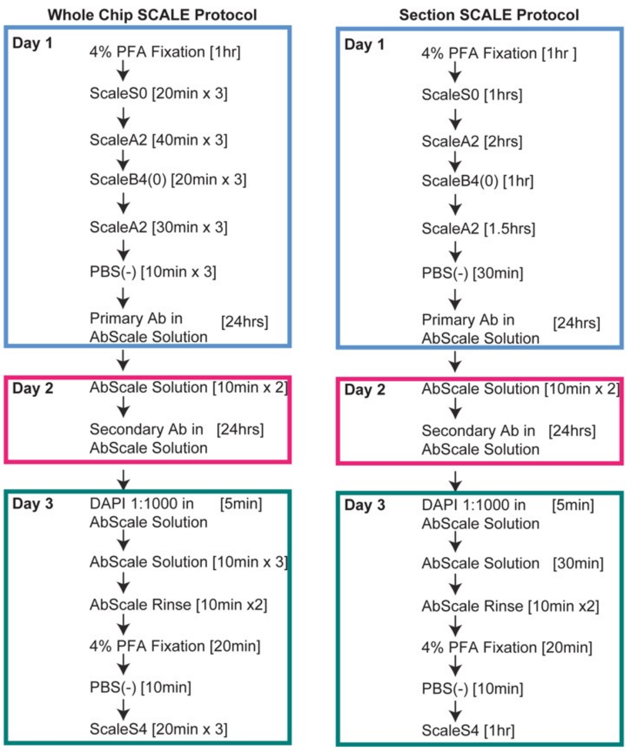

Supplemental Figure 1: Flow diagrams depicting the timing of whole chip SCALE and section SCALE.

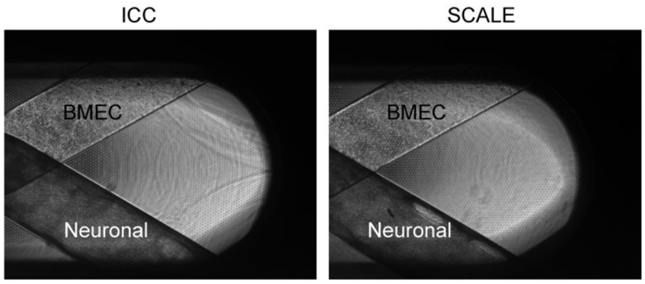

Supplemental Figure 2: Representative phase contrast imaging of d28 chips taken prior to staining or clearing applications. BMEC channel is shown at the top and neuronal channel is indicated at the bottom.
