# Supplementary figures and images for: Tissue clearing of human iPSC-derived organ-chips enables high resolution imaging and analysis
Source: Lab Chip. 2022 Oct 7;22(21):4246–55. doi: 10.1039/d2lc00116k (PMC9595176; doi:10.1039/d2lc00116k)

Supplementary Figure 1

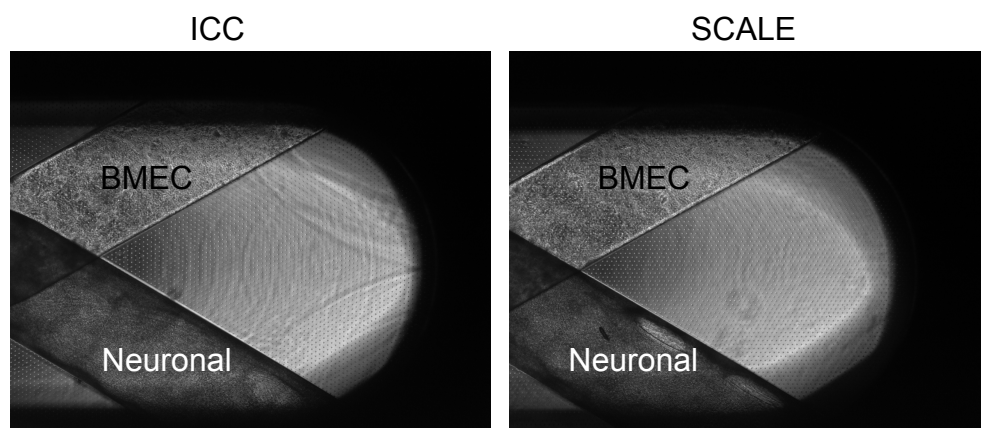

Supplement: LC-022-D2LC00116K-s002 [file LC-022-D2LC00116K-s002.pdf]
